# Alterations in the proteomes of HepG2 and IHKE cells inflicted by six selected mycotoxins

**DOI:** 10.1007/s00204-024-03905-0

**Published:** 2024-12-06

**Authors:** Lucas Keuter, Marco Fortmann, Matthias Behrens, Hans-Ulrich Humpf

**Affiliations:** Institute of Food Chemistry, University of Münster, Corrensstraße 45, 48149 Münster, Germany

**Keywords:** Bioactivity, Mycotoxins, Proteomics, Liver, Kidney, Mode of action, Toxicity pathway

## Abstract

Toxic fungal secondary metabolites, referred to as mycotoxins, emerge in moldy food and feed and constitute a potent but often underestimated health threat for humans and animals. They are structurally diverse and can cause diseases after dietary intake even in low concentrations. To elucidate cellular responses and identify cellular targets of mycotoxins, a bottom-up proteomics approach was used. We investigated the effects of the mycotoxins aflatoxin B_1_, ochratoxin A, citrinin, deoxynivalenol, nivalenol and penitrem A on the human hepatoblastoma cell line HepG2 and of ochratoxin A and citrinin on the human kidney epithelial cell line IHKE. Incubations were carried out at sub-cytotoxic concentrations to monitor molecular effects before acute cell death mechanisms predominate. Through these experiments, we were able to detect specific cellular responses that point towards the mycotoxins’ mode of action. Besides very well-described mechanisms like the ribotoxicity of the trichothecenes, we observed not yet described effects on different cellular mechanisms. For instance, trichothecenes lowered the apolipoprotein abundance and aflatoxin B_1_ affected proteins related to inflammation, ribogenesis and mitosis. Ochratoxin A and citrinin upregulated the minichromosomal maintenance complex and nucleotide synthesis in HepG2 and downregulated histones in IHKE. Penitrem A reduced enzyme levels of the sterol biosynthesis. These results will aid in the elucidation of the toxicodynamic properties of this highly relevant class of toxins.

**Supplementary Information:**

The online version contains supplementary material available at 10.1007/s00204-024-03905-0.

## Introduction

This study evolves around mycotoxins, which are secondary metabolites of different fungal species that can contaminate food and feed. Because of their toxicity to humans and animals, they represent a serious health threat and are partially regulated by legal limits (Khan et al. 2024; Eskola et al. 2020). The basis for such regulations, e.g., in terms of maximum levels in food, is a comprehensive risk assessment (More et al. 2019). A decisive part of risk assessment in the adverse outcome pathway (AOP) framework is the elucidation of toxicity pathways (Ankley et al. 2010), which are defined as “cellular response pathways that, when sufficiently perturbed, are expected to result in adverse health effects” (Krewski et al. 2010). AOPs are a superordinate concept used in risk assessment to characterize the pathway of biological events initiated by toxic compounds to adverse outcomes on the organism or even population level, also without the use of animal tests (Ankley et al. 2010; Allen et al. 2014).

The process of identifying such toxicodynamic properties is not straightforward, since the investigated toxins exhibit different modes of action (MoA) and choosing an appropriate assay sometimes turns into a game of chance. Nevertheless, decades of research identified several distinct toxicity pathways for some of the most relevant mycotoxins (Awuchi et al. 2022). However, research on the bioactivity of mycotoxins was mainly based on targeted assays to investigate certain mechanisms. In 2018, the European Food Safety Authority (EFSA) stated in their Scientific Colloquium 24 that omics techniques can be a valuable addition to identify toxicity pathways in the AOP framework (Aguilera et al. 2018). Furthermore, the EFSA specifically mentioned omics techniques as a tool to characterize MoA of mycotoxins (Beatriz and Nolwenn 2022). The big advantage of such methodologies is the comprehensive investigation of any possible alteration in the considered biological system (Gutierrez Reyes et al. 2024). In addition, Cimbalo et al. (2022) described the usefulness of transcriptomic and proteomic data on the characterization of mycotoxins’ cellular effects and emphasized missing data for several compounds. The big advantage of such untargeted study designs over specific assays is the drastically reduced likelihood of missing otherwise undetected effects.

The depth of knowledge about the different mycotoxins’ toxicity pathways is unevenly distributed. For few compounds, cellular mechanisms were precisely elucidated, while for many others, only adverse effects on certain organs or even less have been reported. For instance, trichothecenes such as deoxynivalenol (DON) and nivalenol (NIV), primarily produced by *Fusarium* spp., are well-known ribotoxins in eukaryotes: They inhibit the protein synthesis by binding to the ribosomal large subunit (LSU, also referred to as 60S) and, therefore, impede the formation of peptide bonds (McCormick et al. 2011; Cundliffe et al. 1974). On the other hand, aflatoxins like aflatoxin B_1_ (AFB_1_), mainly formed by *Aspergillus* spp., are known to induce hepatocellular carcinoma and to act mutagenic and teratogenic (Kensler et al. 2011). Although several studies observed inflammatory effects and oxidative stress induced by these mycotoxins, their precise cellular mechanisms are not fully elucidated yet (Cimbalo et al. 2022; Frangiamone et al. 2024; Wen et al. 2016). For the *Aspergillus* and *Penicillium* spp. derived mycotoxin ochratoxin A (OTA), the production of reactive oxygen species (ROS), damage of deoxyribonucleic acid (DNA), inhibition of protein synthesis and cell cycle arrest are described as impaired cellular processes (Liu et al. 2022; Frangiamone et al. 2024). Citrinin (CIT) is produced by the same fungal genera as OTA and is described to have similar effects such as cell cycle arrest, oxidative and inflammatory stress, but also indications of genotoxic and mutagenic effects through DNA damage (Oliveira Filho et al. 2017). The IARC ranks CIT as “Not classifiable as to its carcinogenicity to humans” in group 3 (IARC 1987). Research on the bioactivity of penitrem A (Pen A) is very scarce. The tremorgenic mycotoxin is produced by certain *Aspergillus*, *Claviceps* and *Penicillium* spp. and is described to be cytotoxic and to affect amino acid homeostasis and urea cycle in HepG2 cells (Kalinina et al. 2018; Gerdemann et al. 2022). Furthermore, the production of ROS in human neutrophils was observed (Berntsen et al. 2017).

Our study aims to identify alterations in the proteome of HepG2 cells that allow to characterize the main cellular targets of mycotoxins and to investigate their MoA. We selected the six mycotoxins AFB_1_, OTA, CIT, DON, NIV, and Pen A (structures in Online Resource 1, Figure S1) that are either regulated, relevant because of high occurrence, or showed noteworthy behavior in previous experiments. The human hepatoblastoma cell line HepG2 was used to mimic the liver as the main organ of xenobiotic metabolism, in accordance with a previous metabolic profiling study (Gerdemann et al. 2022). Moreover, the nephrotoxic compounds OTA and CIT were applied to immortalized human kidney epithelial cells (IHKE, Schwerdt et al. 1999; Oliveira Filho et al. 2017). Due to proposed combinatory effects, a mixture of both mycotoxins was used as well (Schulz et al. 2018; Knecht et al. 2005). The cells were treated with sub-cytotoxic concentrations, insignificantly impairing the overall cellular viability (maximum decrease in cell viability of 20%), based on previous assays on the cellular redox activity status (Gerdemann et al. 2022; Zingales et al. 2020; Kalinina et al. 2018; Knecht et al. 2005; Bittner et al. 2015).

## Materials and methods

This section is based on the reporting guidelines for proteomics experiments of the Human Proteome Organization (HUPO, Taylor et al. 2007). Chapters on chemicals and reagents, cell culture and sample preparation (based on the filter-aided sample preparation method by Wiśniewski et al. 2009 and as previously described in Müller et al. 2022) are depicted in Online Resource 1.

### Cell treatment

1 × 10^6^ HepG2 cells or 2 × 10^6^ IHKE cells were seeded onto cell culture dishes of 3.5 cm or 6 cm diameter, respectively. After 48 h of growth, cell culture medium was replaced with serum-free medium supplemented with buffer and antibiotics for further 24 h. Afterwards, the cell culture medium was replaced with mycotoxins in different concentrations (Table 1) in fresh serum-free medium and incubated for 24 h.

**Table 1:** Concentrations of mycotoxins used for treatment of the liver cell line HepG2 and the kidney cell line IHKE, each for 24 h and respective literature providing cytotoxicity data for choosing these concentrations

HepG2			IHKE		
AFB_1_	10 µM	Gerdemann et al. (2022)	CIT	15 µM	Knecht et al. (2005)
CIT	20 µM	Gerdemann et al. (2022)	OTA	20 nM	Bittner et al. (2015)
DON	1 µM	Gerdemann et al. (2022)	CIT + OTA	15 µM + 20 nM	
NIV	0.5 µM	Zingales et al. (2020)			
OTA	200 nM	Gerdemann et al. (2022)			
Pen A	10 µM	Kalinina et al. (2018)			

AFB_1_, aflatoxin B_1_; NIV, nivalenol; DON, deoxynivalenol; Pen A, penitrem A; CIT, citrinin; OTA, ochratoxin A

In case of AFB_1_, an additional experiment with metabolically induced HepG2 cells was performed. Therefore, cells were pretreated with 10 µM β-naphthoflavone (β-NF) in serum-free medium 16 h prior to mycotoxin treatment. In addition, corresponding solvent controls treated with 1% acetonitrile (ACN) were added to the experiments. Three independent cell passages were used, each with two replicates prior to cell seeding (*n* = 3 × 2).

### HPLC–MS

Samples were analyzed using an Elute high-performance liquid chromatography (HPLC) pump coupled to an Impact™ II quadrupole time-of-flight (QTOF) mass spectrometer equipped with an Apollo II source (Bruker, Bremen, Germany). 45 µL was injected onto a Peptide Mapping 2.1 × 150 mm column with 120 Å pore size and 2.7 µm particle size (Agilent, Santa Clara, CA, USA). The HPLC gradient of acetonitrile and water both with 0.1% formic acid had 100 min of active gradient time and the mass spectrometer was operated in data-dependent acquisition (DDA) mode. Detailed method information is depicted in the Online Resource 1, Table S1.

### Data processing

Proteins were identified and quantified in a label-free quantification (LFQ) approach using MaxQuant version 2.4.3 and processed by default values if not specified otherwise (Cox et al. 2014; Cox and Mann 2008). Only reviewed human proteins (Uniprot proteome: UP000005640, accessed 2 May 2023) were used for identification. False discovery rate (FDR) was set to 1% for both proteins and peptides. Methionine oxidation and protein *N*-terminal acetylation were allowed as variable modifications. LFQ with classic normalization and the fast LFQ option with a minimum of 5 and an average of 8 neighbors was performed. Default Bruker QTOF values were used as instrument parameters in MaxQuant. Only unmodified peptides were allowed for quantification.

### Statistical analysis

The “proteinGroups.txt” file from MaxQuant was uploaded to Perseus version 2.0.11. Gene Ontology (GO) and Kyoto Encyclopedia of Genes and Genomes (KEGG) annotations were added (accessed 1 April 2024). Proteins identified by site, reverse proteins and potential contaminants were removed. LFQ intensities were transformed by log_2_(x) and replicates were grouped. An experiment was defined as the combination of all replicates of treated samples of one mycotoxin with respective controls. Volcano plots of each experiment were generated by plotting -log_10_(*p-*value) against the difference of logarithmized LFQ intensities for each protein between treated sample and control, referred to as log_2_ fold change (log_2_ FC) in the following. Significance of protein abundance change was determined by *t*-test and a permutation-based FDR. Proteins were considered as differentially abundant between samples, if it met the criteria of Perseus’ two-sided *t*-test with 250 randomizations, FDR ≤ 0.05 and *S*0 = 0.1. The number of differentially abundant proteins (DAPs) was identified at this point.

### Interaction and enrichment analysis

Whole proteome data of each experiment containing protein identifiers and log_2_ fold change (FC) values between sample and control were uploaded in the “proteins with values/ranks” function of STRING DB version 12.0 (string-db.org, Szklarczyk et al. 2023) and analyzed for functional enrichments. In case of protein groups, only the first accession was used for these functional analyses. STRING annotates proteins with database terms, e.g., GO annotations and KEGG pathways and performs a functional enrichment analysis by calculating an enrichment score (ES). Through this calculation, the significant enrichment of a group of proteins with a common biological function regarding GO or KEGG terms to the down-, upregulated or to both sides is analyzed. To calculate the ES, the mean log_2_ FC of proteins of a certain term is calculated at first (mean of term). Second, the mean log_2_ FC of the whole input set (mean of input) is subtracted from this mean of term (mean of term − mean of input). The ES is then calculated as the ratio between “mean of term − mean of input” and the maximum log_2_ FC of the input set, if mean of term > mean of input, or the minimum log_2_ FC, if mean of term < mean of input, multiplied by 10 (Szklarczyk et al. 2019). The expected proportion of false-positive identifications, the FDR, is calculated based on either the aggregate fold change model (Yu et al. 2017) or two-sided Kolmogorov–Smirnov testing, which depends on the size of each term and its deviation from the mean. Bubble plots of the top functional enrichments containing term description, ES of the term, FDR, and percentage of quantified proteins of the term were generated in SRplot (Tang et al. 2023).

In addition, DAPs were picked from Perseus-generated volcano plots and uploaded as “multiple proteins” into STRING DB and analyzed for functional enrichments and protein–protein interactions. All active interaction sources were allowed here and a medium confidence of 0.4 was set as required interaction score. Background proteomes of HepG2 and IHKE cells were generated from deep proteome analysis approaches using high pH reversed phase fractionation with concatenation modified from Wang et al. (2011) extended by proteins identified in other in-house shotgun proteomics analyses of the respective cell lines. These proteomes were used as statistical backgrounds for interaction analysis in DAPs in STRING (protein lists in Online Resource 2).

## Results and discussion

The six mycotoxins AFB_1_, OTA, CIT, DON, NIV, and Pen A were applied to the human hepatoblastoma cell line HepG2 and CIT, OTA and a combination of both on the human kidney epithelial cell line IHKE in sub-cytotoxic concentrations. The results are described based on two evaluation methods both assisted by STRING DB.

First, bubble enrichment plots display the significant enrichment of protein groups with a common biological function to the down-, upregulated or both sides of a whole proteome (comparable to gene set enrichment analysis, for detailed information see chapter Interaction analysis). In this case, the biological function is described as terms of Gene Ontology (GO) annotations, which contain biological processes (BPs), cellular components (CCs) and molecular functions (MFs) as well as Kyoto Encyclopedia of Genes and Genomes (KEGG) terms, which contain important signaling and metabolic pathways. The bubble plots presented here show only the strongest effects (highest enrichment scores, ESs) of each experiment on the cellular proteomes. The ES describes how distant the specific, term-associated proteins are from the middle of the proteome. In other words, the ES characterizes the intensity of a deregulation of a certain biological function, expressed in the form of up- or downregulated proteins. It should be noted that ESs for bubble plots are calculated individually for each experiment (see chapter Interaction analysis) and for this reason, the scale of the x-axis is not comparable. In addition, FDR scale and size of bubbles, representing the identified percentage of a term, are individual per plot. However, all shown effects have an FDR < 0.01.

Second, functional enrichments within the group of DAPs from either side were identified and taken into account for interpretation. In this case, not the whole proteome data were used, but only the interactions between significantly altered proteins from either side were analyzed—regardless of their fold change. These results are shown in the Online Resources 3 (HepG2) and 4 (IHKE).

All proteomic effects of the investigated mycotoxins are provided in Online Resources 3 and 4—individually per protein and as enriched terms by STRING analyses. Additional to enriched terms, changes in the abundance of individual proteins were considered. From all these effects, the main cellular targets of the mycotoxins’ toxicity were derived and potential MoA were illustrated.

The overall number of altered proteins in the HepG2 proteome varied according to the incubated mycotoxin. In terms of amount of DAPs, DON had the strongest effect, as it induced significant changes for 17% of all proteins (see Online Resource 1, Figure S2), according to the Perseus analysis (see chapter Statistical analysis). OTA and NIV had comparable effects, with 6.7% and 5.3%, respectively. In comparison, Pen A, CIT and AFB_1_ showed fewer significant alterations, with 1.2%, 0.80% and 0.53%, respectively, but the overall effect of AFB_1_ was increased by the pretreatment of HepG2 cells with β-NF to 3.2%. In IHKE cells, OTA also had a stronger overall effect on the proteome, with 14% DAPs, than CIT, with 0.62%. Their combination led to 2.3% of DAPs in the IHKE proteome. The following sections provide an overview of the effects caused by the individual mycotoxins.

### Ochratoxin A

**Fig. 1:**
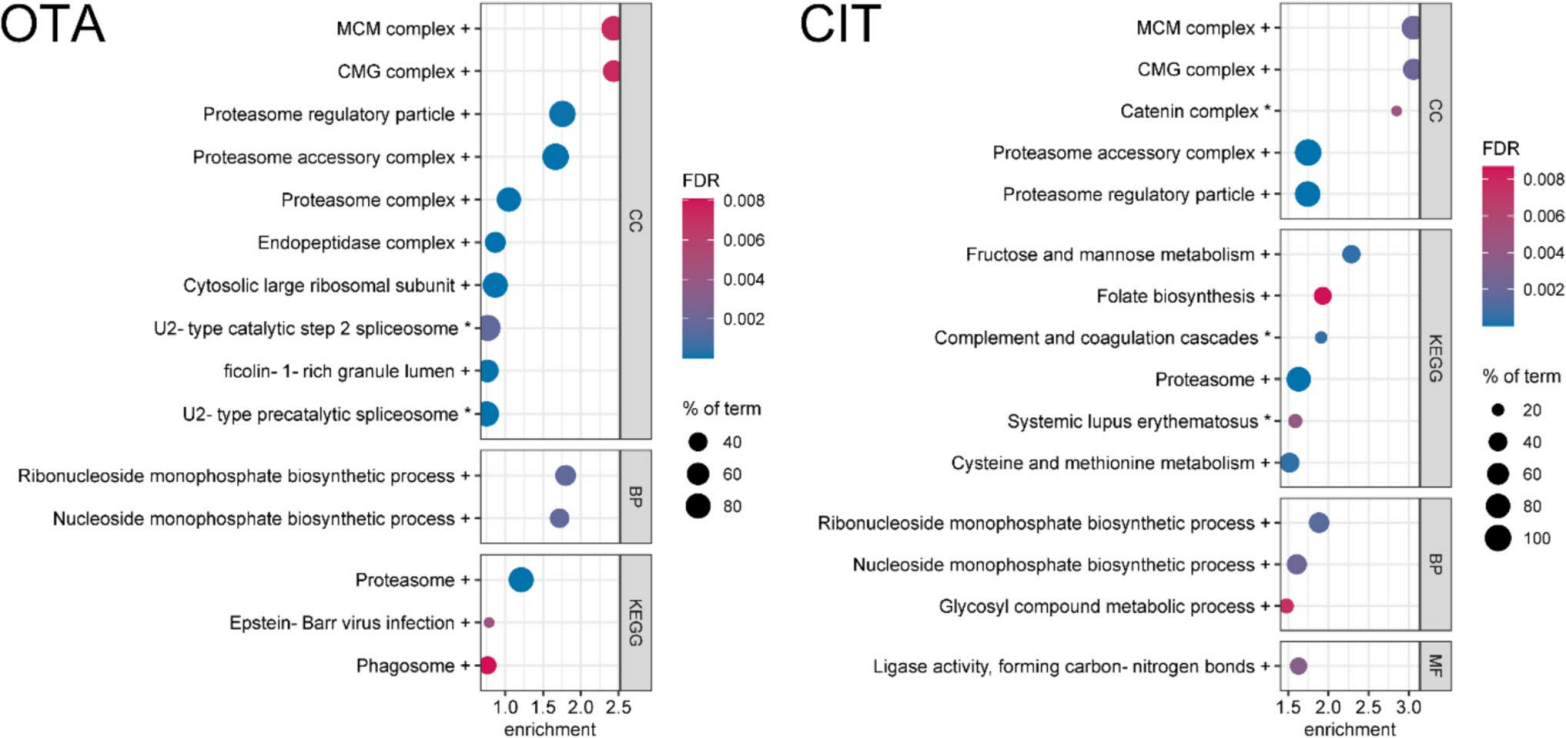
Enrichment bubble plots of the top 15 enriched terms in the whole proteome of HepG2 cells treated with ochratoxin A (OTA, 200 nM) and citrinin (CIT, 20 µM) for 24 h. Effects on cellular components (CC), biological processes (BP), molecular functions (MF, all from Gene Ontology) and terms of the Kyoto Encyclopedia of Genes and Genomes (KEGG) are shown. The color scale describes the significance as the false discovery rate (FDR) and the bubble size scales with the percentage of identified proteins affiliated to the described term. Terms are annotated by + for upregulation and * for downregulation. Enrichment analysis was performed by STRING DB

HepG2 cells were treated with 200 nM OTA for 24 h (Fig. 1, left). Strongest effects were observed in the upregulation of the “MCM^1^ complex”, which is a heterohexamer controlling DNA replication in the late M to early G_1_ phase of the cell cycle (Lei 2005). The “CMG^2^ complex” showed the same values as the “MCM complex” of ES 2.43 and an FDR of 0.0074. These two terms include the same six modulated proteins (MCM2–MCM7), but three CMG-specific proteins were not identified. These results were supported by enriched terms associated with DNA replication and the MCM complex itself in the group of upregulated DAPs. The deregulation of the MCM complex is associated with the development of hepatocellular carcinoma and genomic instability (Lei et al. 2021). MCM plays a key role in DNA replication—replicative stress is discussed as a potential cause for genotoxic properties of OTA. However, the underlying mechanism remains unclear (EFSA Panel on Contaminants in the Food Chain (CONTAM) et al. 2020; Klotz et al. 2022). Our results support the mechanism of DNA replication as a relevant target of OTA toxicity, as the upregulation of the MCM complex was the strongest enriched term. Potentially, cells counter-regulate the inhibitory effect of OTA on DNA replication by upregulation of the MCM complex.

The BPs on ribonucleoside and nucleoside monophosphate biosynthesis were upregulated with ESs of 1.80 and 1.72, respectively, and FDRs of 0.0016, both, likewise supported by the DAP results (see Online Resource 3). The upregulation of proteins involved in nucleotide biosynthesis could be an indirect effect of the disturbed DNA replication, for which a balanced nucleotide pool is required. The induction of respective genes might be regulated via the transcription factor c-Myc, as there is a high overlap between its regulated genes, the proteins upregulated by OTA and those affiliated to nucleoside monophosphate biosynthesis (Liu et al. 2008). Liang et al. (2015) also described the deregulation of nucleotide metabolism and cell cycle as well as DNA repair mechanisms and the blockade of RNA synthesis by OTA in human embryonic kidney cells (HEK293), which mostly supports our results. They concluded that OTA activated the apoptosis signal-regulating kinase 1 (ASK1) via oxidative stress, which in turn led to apoptosis initiation by the mentioned mechanisms.

Several terms of the whole proteome data containing upregulated proteasomal proteins were shown to be enriched with ESs from 1.75 for the CC “proteasome regulatory particle” (FDR < 0.0001) to 1.05 for the CC “proteasome complex” (FDR < 0.0001). The proteasome is a multisubunit protein complex that is responsible for the intracellular degradation of proteins. Its upregulation might be caused by direct effects of OTA or might represent an unspecific stress response. On the one hand, the Nrf2 pathway is described to induce the proteasome as a reaction to oxidative stress (Pickering et al. 2012), which is reported to be a key mechanism of OTA toxicity (Frangiamone et al. 2024). On the other hand, OTA could directly bind to proteins, like described for human and murine serum albumin (Sueck et al. 2018; Kuhn et al. 2024). Malfunctioning modified proteins need to be degraded, which could induce the upregulation of the proteasome. Furthermore, Perugino et al. (2024) recently proposed the inhibition of a prolyl 3-hydroxylase involved in protein synthesis, by 3-dimensional modeling. This effect might induce protein damage resulting in an increased requirement for degradation. Akpinar et al. (2019) also observed a time-dependent deregulation of proteasomal proteins in human kidney proximal tubule cells (HK-2) caused by 10 µM OTA. Furthermore, within the upregulated DAPs, different extracellular components were identified. Effects with ESs < 1 are not further discussed.

In IHKE cells, OTA induced a strong downregulation of several histones and high mobility group nucleosome-binding proteins, which resulted in the enriched MFs “nucleosomal DNA binding” and “structural constituent of chromatin” in the whole proteome data and within the downregulated DAPs (see Online Resource 4). Histone downregulation can be caused by the G_1_ checkpoint pathway, which in turn can be activated by DNA damage (Su et al. 2004). On the upregulated side, mainly proteins of extracellular components and various metabolic processes were enriched (see Online Resource 4).

Taking results from both cell lines together, OTA seems to affect the cell cycle, which is already described (Kőszegi and Poór 2016). Different responses of the two cell lines could be explained by very different methods used to obtain the cells (López-Terrada et al. 2009; Tveito et al. 1989) and by eventually high differences in abundances of proteins like the tumor suppressor p53 in cancer cells (Zhou and Elledge 2000).

### Citrinin

HepG2 cells were treated with 20 µM CIT for 24 h (Fig. 1, right). Similar to the effects of OTA, CIT strongly induced the six MCM subunits MCM2–MCM7 resulting in an ES of 3.06 and an FDR of 0.0022 for the CC terms “MCM complex” and “CMG complex”. This effect is supported by respective terms enriched in the DAPs and several terms regarding DNA replication (see Online Resource 3). Remarkably, the same terms of upregulated proteasomal proteins and nucleotide biosynthesis-related proteins as for OTA were observed to be enriched by CIT as well. For proteasomal proteins, ESs ranged from 1.75 for the CC “proteasome accessory complex” (FDR < 0.0001) to 1.43 for the CC “proteasome complex” (FDR < 0.0001, not part of top 15 terms). Concerning nucleotide biosynthesis, the BP “ribonucleoside monophosphate biosynthesis” showed an ES of 1.88 with an FDR of 0.0014 and the respective nucleoside term showed an ES of 1.61 with an FDR of 0.0022. Supporting these results, terms on the MF “nucleotide binding” were enriched in the analysis of upregulated DAPs (see Online Resource 3). Concerning the MCM upregulation and induction of the proteasome and the nucleotide biosynthesis, CIT showed effects on the proteome of HepG2 cells comparable to OTA (see chapter Ochratoxin A). We assume that this observation indicates a similarity in their toxicity pathways in terms of replication and oxidative stress. A comparison of the log_2_ FC values of the six upregulated MCM proteins between the OTA and the CIT experiment by Student’s *t* test revealed a *p*-value of 0.931, demonstrating a resemblance between the mentioned effects of OTA and CIT. Oxidative stress is a comprehensively analyzed mechanism of CIT and OTA toxicity (Rašić et al. 2019), but replication stress is only discussed for OTA so far (EFSA Panel on Contaminants in the Food Chain (CONTAM) et al. 2020). Thus, the described results reveal a new potential mechanism of CIT toxicity and concurrently suggest the polyketide-derived coumarin part of the molecules (Geisen et al. 2018) as responsible for this mechanism.

The second strongest enriched term was the “catenin complex” (ES 2.85, FDR 0.0045), caused by the downregulation of cadherins (CDH) 1 and 2, catenins α−1 and δ−1 and junction plakoglobin, all of which are junction proteins. As this term was headed by CDH1 (log_2_ FC = − 2.45, − log_10_ *p *value = 0), which was identified in only one out of six replicates of CIT treatment, this effect is not discussed in more detail.

Proteins of the KEGG terms “fructose and mannose metabolism” (ES 2.29, FDR 0.00046) were upregulated, which could affect energy production from glycolysis, but also ascorbate metabolism and *N*-glycan biosynthesis are associated with this pathway (KEGG: hsa00051). On the other hand, some proteins affiliated to “complement and coagulation cascades” (ES 1.91, FDR 0.00052) were downregulated, which were mainly complement factors, serine protease inhibitors and fibrinogens (see Online Resource 3). This could affect the blood coagulation in vivo (Amara et al. 2008). However, no such effects for CIT have been described in the literature.

Enzymes of the “folate biosynthesis” were upregulated (ES 1.93, FDR 0.0087), which could explain the strong enrichment of dihydrobiopterin observed in HepG2 cells (Gerdemann et al. 2022). This effect might be related to nucleotide synthesis—and thereby probably to replication stress—as the output of folate metabolism also includes nucleotide precursors (Zheng and Cantley 2019). Gerdemann et al. (2022) also postulated the inhibition of the enzymes pyruvate carboxylase (PYC) and succinyl-CoA ligase (SUCL), which are part of the citrate cycle. Our results might also explain this result, as PYC (log_2_ FC − 0.743, − log_10_ *p* value 1.29) and SUCL (log_2_ FC − 0.646, − log_10_ *p *value 1.33 for subunit G2) were the strongest downregulated proteins of the enriched KEGG term “citrate cycle” (ES 1.43, FDR 0.0034) that was not listed within the top 15 terms.

In IHKE cells, only the CC “mitochondrial protein-containing complex” and the KEGG term “systemic lupus erythematosus” were enriched in the whole proteome data (see Online resource 4). The latter term was mainly driven by downregulation of histones, which was comparable to OTA. However, the histone downregulation still indicates a similarity in their MoA, which is supported by observations in both HepG2 and IHKE cells.

The experiment with a combination of CIT and OTA did not show any effects that point towards specific combinatory effects on the proteome in the used concentrations of 15 µM and 20 nM, respectively, beyond the addition of the single compound effects—based on the enriched terms in the whole proteome data (see Online Resource 4). In contrast, the combination showed a smaller number of DAPs than OTA alone (see Online Resource 1, Figure S2). This could be caused by the inhibitory effect of CIT on the uptake of OTA described by Knecht et al. (2005). They described, that 15 µM CIT reduced the uptake of OTA by more than 60% in IHKE cells.

### Aflatoxin B_1_

**Fig. 2:**
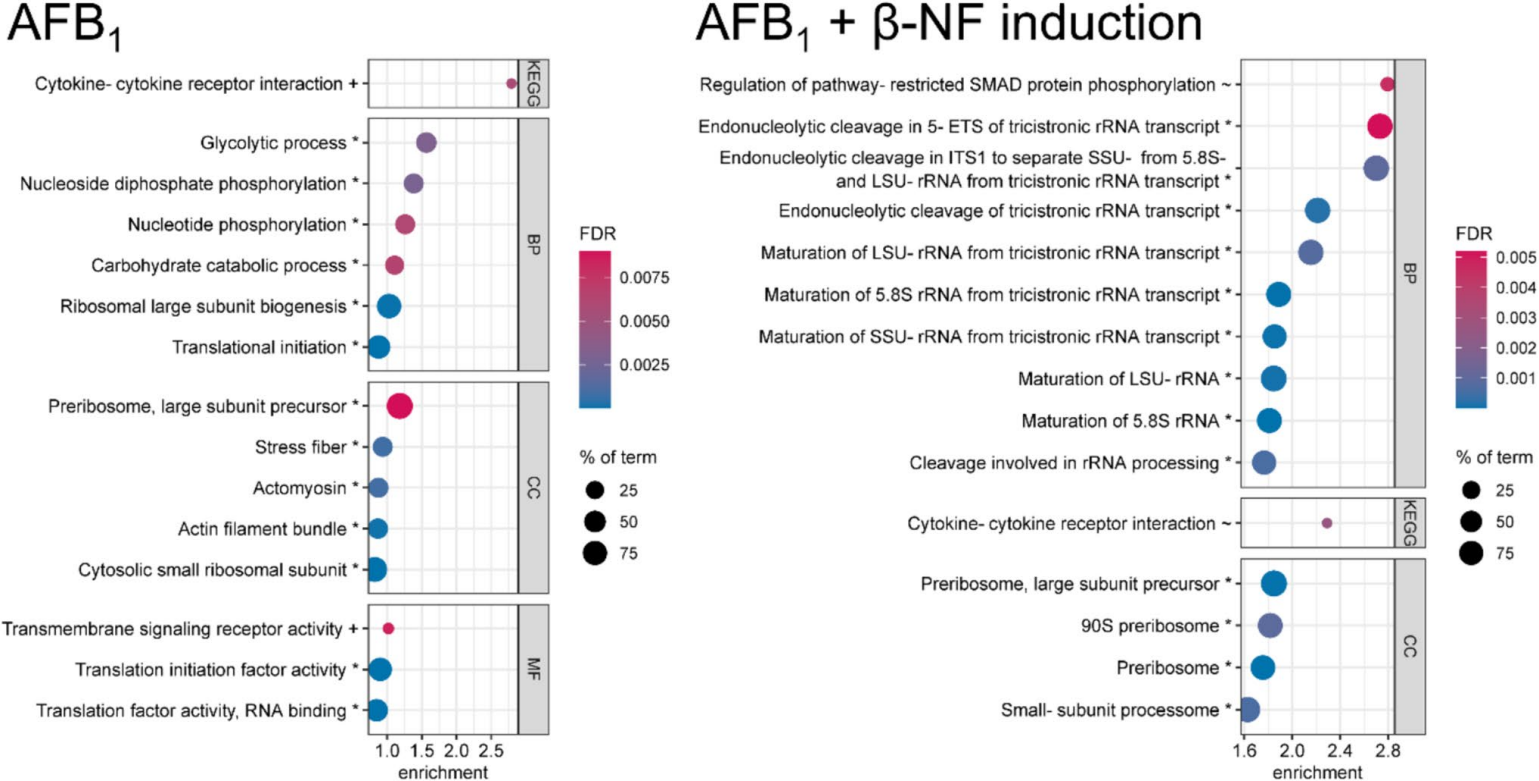
Enrichment bubble plots of the top 15 enriched terms in the whole proteome of HepG2 cells treated with aflatoxin B1 (AFB_1_, 10 µM) for 24 h without (left) or with previous metabolic induction by 10 µM β-naphthoflavone (β-NF, right). Effects on cellular components (CC), biological processes (BP), molecular functions (MF, all from Gene Ontology) and terms of the Kyoto Encyclopedia of Genes and Genomes (KEGG) are shown. The color scale describes the significance as the false discovery rate (FDR) and the bubble size scales with the percentage of identified proteins affiliated to the described term. Terms are annotated by + for upregulation, * for downregulation and ~ for enrichment on both ends. Tricistronic rRNA transcript describes the SSU-rRNA, 5.8S rRNA, LSU-rRNA variant. Enrichment analysis was performed by STRING DB

HepG2 cells were treated with 10 µM AFB_1_ for 24 h (Fig. 2, left). Since the metabolic activation of AFB_1_ was shown to be critical for certain toxic mechanisms (Gerdemann et al. 2023; van Vleet et al. 2002), the same experiment was additionally conducted in β-NF pretreated HepG2 cells (10 µM, 16 h) to induce the metabolic activity especially of CYP1A variants (Westerink and Schoonen 2007; Gerets et al. 2012). In this study, only CYP1A1 was observed as induced (see Online Resource 3, sheet “β-NF proteins”), but CYP1A2 was shown to be induced by β-NF in HepG2 cells in former investigations (data not shown) and presumably lacks abundance to meet the limit of detection. The latter experiment included its own corresponding control pretreated with β-NF and afterwards treated with solvent control (see chapter Cell treatment). The results are shown in the right-hand part of Fig. 2.

For AFB_1_, by far the strongest enriched term was the “cytokine–cytokine receptor interaction” (ES 2.79, FDR 0.0057), mainly caused by the upregulated cytokine “growth and differentiation factor 15” (GDF15, log_2_ FC 1.72, − log_10_ *p* value 4.87), but also by tumor necrosis factor receptors and interleukin receptors (see Online Resource 3). In β-NF pretreated cells, again the KEGG term “cytokine–cytokine receptor interaction” was found enriched (ES 2.29, FDR 0.0026) with the same proteins involved, but in this case, enrichment on both ends (up- and downregulation) was identified. Like for AFB_1_ without pretreatment, GDF15 was the main driver for this term (log_2_ FC 3.72, − log_10_ *p* value 4.27), but also affected the enriched BP term “regulation of pathway-restricted SMAD protein phosphorylation” (ES 2.79, ES 0.0046). The strong upregulation of GDF15, even enhanced by pretreatment with β-NF to increase metabolic activation of AFB_1_, probably indicates an inflammatory response or other cellular dysfunctions (Wang et al. 2021). GDF15 is used as a biomarker for cardiovascular disease, cancer and other diseases in humans (Luan et al. 2019). Therefore, the effect of AFB_1_ on GDF15 abundance in vivo should be analyzed to prevent false-positive diagnosis of these diseases. Our results indicate that the metabolic activation of AFB_1_ through phase I metabolism is required for the inflammatory response in HepG2 cells or at least induces it. The hypothesis of inflammatory processes is supported by respective enriched terms that include GDF15 as well as different cytokine receptors. Except for GDF15, no cytokine was detected, which could be related to their low molecular weight and subsequent loss during sample preparation. During inflammatory processes, interleukins are excreted to cell culture media which was removed prior to sample preparation and, therefore, not analyzed in this study. Eventually, the abundance of cytokine receptors could have been upregulated because of a high, but not detectable cytokine concentration. Iori et al. (2022) observed the same KEGG term “cytokine–cytokine receptor interaction” as strongest enriched in a transcriptomic approach in bovine liver cells. They proposed an activation of the toll-like receptor 2 linked to inflammatory response and oxidative stress. Other transcriptomic studies using the chicken hepatocellular carcinoma cell line LMH and the bovine fetal hepatocyte cell line BFH12 found impaired genes associated with inflammation as well (Choi et al. 2020; Pauletto et al. 2020).

Within the upregulated DAPs caused by AFB_1_ in non-pretreated cells, several terms related to the mitotic cell cycle were identified as enriched, although only 13 proteins (0.43% of all quantified) were identified as significantly upregulated (see Online Resource 3). Within these terms, the aurora kinases A and B (AURKA, log_2_ FC 0.63, − log_10_ *p* value 3.28; AURKB, log_2_ FC 0.75, − log_10_ *p* value 3.04) and the inner centromere protein (INCENP, log_2_ FC 0.76, − log_10_ *p* value 3.53) were the key proteins. Aurora kinases and INCENP are parts of the chromosomal passenger complex and play a central regulatory role in mitosis and cytokinesis. The deregulation of these processes could lead to the described general cytotoxicity (Cimbalo et al. 2022), but could also induce chromosomal defects (Ruchaud et al. 2007) and thereby contribute to the carcinogenicity of AFB_1_.

Further enriched terms in the whole proteome dataset of only AFB_1_ describe the downregulation of enzymes involved in glycolysis or nucleotide phosphorylation, with a high overlap within the proteins of these terms: all proteins of the BP “glycolytic process” were also found in the BP “nucleotide phosphorylation” (see Online Resource 3). The downregulation of these enzymes matches the decreased concentration of nucleoside derivatives and several metabolites of glycolysis found after AFB_1_ treatment in HepG2 cells (Gerdemann et al. 2022).

All other top terms of AFB_1_ treatment in metabolically induced HepG2 cells describe downregulated proteins of processes or components of the maturation of rRNA or ribosomes. For instance, the BP “endonucleolytic cleavage in 5-ETS of tricistronic rRNA transcript (SSU-rRNA, 5.8S rRNA, LSU-rRNA)” was enriched by ES 2.73 with an FDR of 0.0052. None of these terms occurred in the top 15 enriched terms in non-pretreated cells. The ribosome biogenesis and its related terms were very much represented in the enriched terms within the group of downregulated DAPs as well (see Online Resource 3) and precursors of the ribosomal LSU. The CC “preribosome, large subunit precursor” was already found in the experiment with non-pretreated cells. However, the high abundances and ESs of terms related to ribosome biogenesis or, more specifically, rRNA maturation indicate higher effects after metabolic induction. This suggests that the activation of AFB_1_ through phase I metabolism enhances its effect on these terms. However, no such effect on ribosomes or ribosomal activity is described for AFB_1_ so far, except for the aforementioned transcriptomics approach by Iori et al. (2022), who found the term “ribosome biogenesis in eukaryotes” enriched. These results point towards a new potential cellular target of AFB_1_ that could contribute to its hepatotoxicity. Whether the downregulation of ribosomal proteins actually impairs their activity in the form of protein synthesis needs to be investigated in further experiments. Effects with ESs < 1 are not discussed in detail.

### Penitrem A

**Fig. 3:**
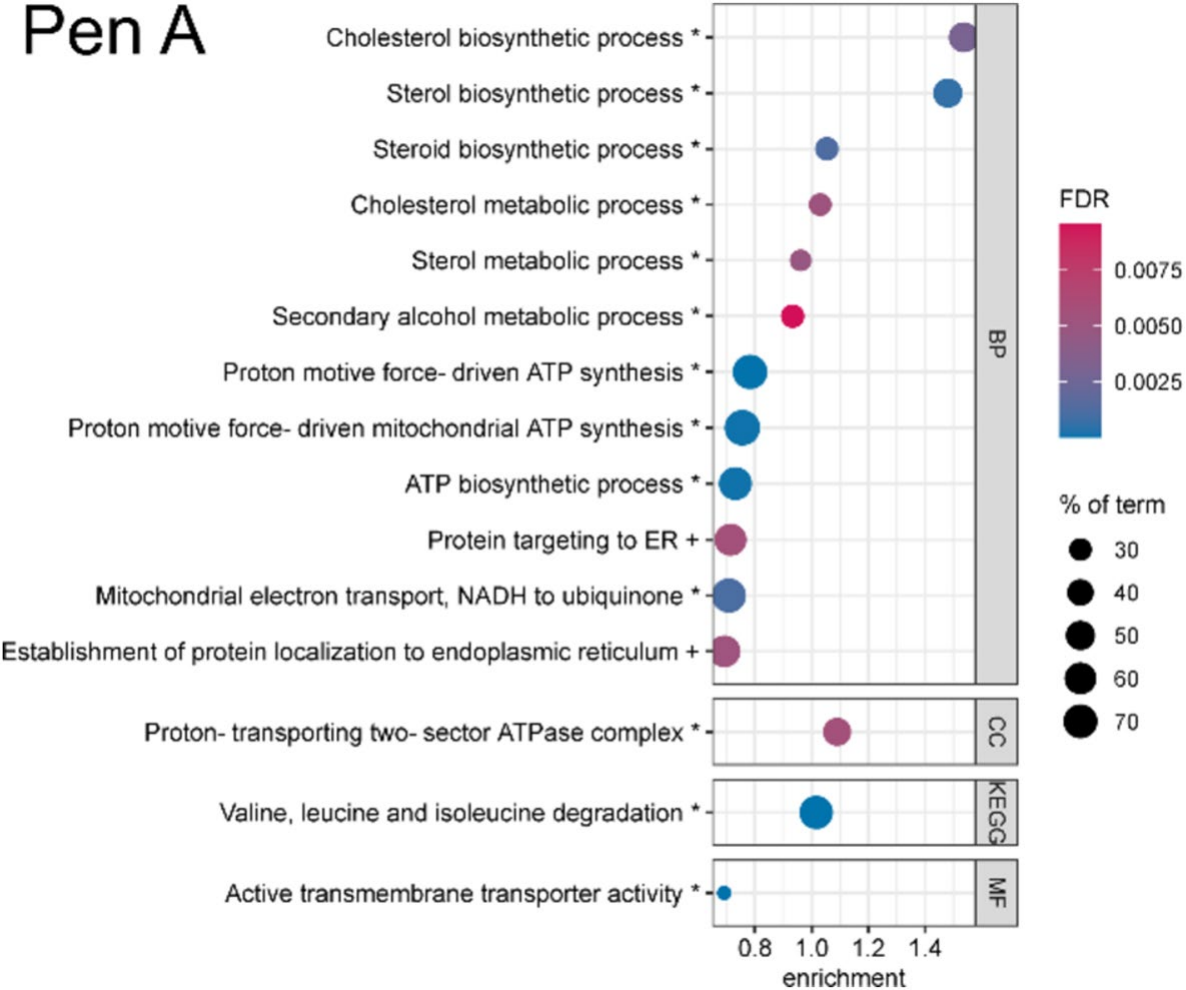
Enrichment bubble plots of the top 15 enriched terms in the whole proteome of HepG2 cells treated with penitrem A (Pen A, 10 µM) for 24 h. Effects on cellular components (CC), biological processes (BP), molecular functions (MF, all from Gene Ontology) and terms of the Kyoto Encyclopedia of Genes and Genomes (KEGG) are shown. The color scale describes the significance as the false discovery rate (FDR) and the bubble size scales with the percentage of identified proteins affiliated to the described term. Terms are annotated by + for upregulation and * for downregulation. Enrichment analysis was performed by STRING DB

HepG2 cells were treated with 10 µM Pen A for 24 h. The enrichment analysis of the few DAPs (1.2%) revealed no enriched GO or KEGG terms in these groups. However, the analysis of the whole proteome dataset revealed significant enrichments of certain biological functions (Fig. 3). The strongest effect was observed in the downregulation of proteins involved in the “cholesterol biosynthetic process” (ES 1.54, FDR 0.0030), while several other terms containing “sterol”, “steroid” or “secondary alcohol” showed a high overlap with this term. The diterpene part of the chemical structure of Pen A might cause the downregulation of proteins involved in these processes, as it comprises a similarity to the polycyclic backbone of cholesterol. Comparably, the exogenous steroid hypocholamide regulates the expression of genes involved in cholesterol and fatty acid homeostasis via the liver X receptor (Song and Liao 2001). Potentially, Pen A activates negative regulatory feedback pathways of cholesterol synthesis and metabolism by binding to this receptor. Inhibited synthesis of cholesterol in the liver in vivo can affect the uptake, metabolism and transport of lipids, but also more severe effects on the entire organism are described, especially during developmental stages (Peeples et al. 2024).

Another term contains downregulated proteins of the “proton-transporting two-sector ATPase complex” (ES 1.09, FDR 0.0056), of which 24 different ATPases or subunits were identified. These also led to the enriched terms concerning proton motive force-driven ATP synthesis. The effect on mitochondrial ATP synthesis was highly specific, as almost all ATPases or subunits were downregulated. Its relevance for cellular energy levels is apparent and could explain cytotoxic effects of Pen A in higher concentrations (Gerdemann et al. 2022; Kalinina et al. 2018). A third effect was the downregulation of valine, leucine and isoleucine degrading enzymes (ES 1.02, FDR < 0.0001). The deregulation of branched-chain amino acid degradation via transamination and oxidative decarboxylation is associated with obesity, insulin resistance and diabetes (Choi et al. 2024).

Few studies analyzed cellular toxicity pathways of Pen A so far and these focused on cytotoxicity (Kalinina et al. 2018), metabolic (Gerdemann et al. 2022) or tremorgenic effects in the central nervous system (Berntsen et al. 2013). Our study suggests three new potential deregulated mechanisms by Pen A, which are sterol biosynthesis and metabolism, mitochondrial energy production and branched-chain amino acid degradation. These results can contribute to further understand the detailed mechanisms behind the (cyto)toxicity of Pen A. Future studies should include the investigation of proteomic alterations in cells of the central nervous system, the main site of action of Pen A toxicity in vivo. For this purpose, e.g., CCF-STTG1 could be used, in which Pen A has shown stronger cytotoxicity than in HepG2 cells (Kalinina et al. 2018).

### Trichothecenes

For the two type B trichothecenes DON and NIV, a deviating presentation of the enrichment analysis results was chosen. Due to their well-described ribotoxicity, effects on the abundance of ribosomal proteins were expected. These were observed in the form of upregulated proteins involved in ribosomal biogenesis. However, the corresponding enriched terms were the strongest ones on the upregulated side, but not within the overall top 15 enriched terms, since the terms on the downregulated side showed much higher ESs. As we still intended to demonstrate the ribotoxicity-related effects of trichothecenes, the following bubble plots are divided in up- and downregulated terms.

### Deoxynivalenol

**Fig. 4:**
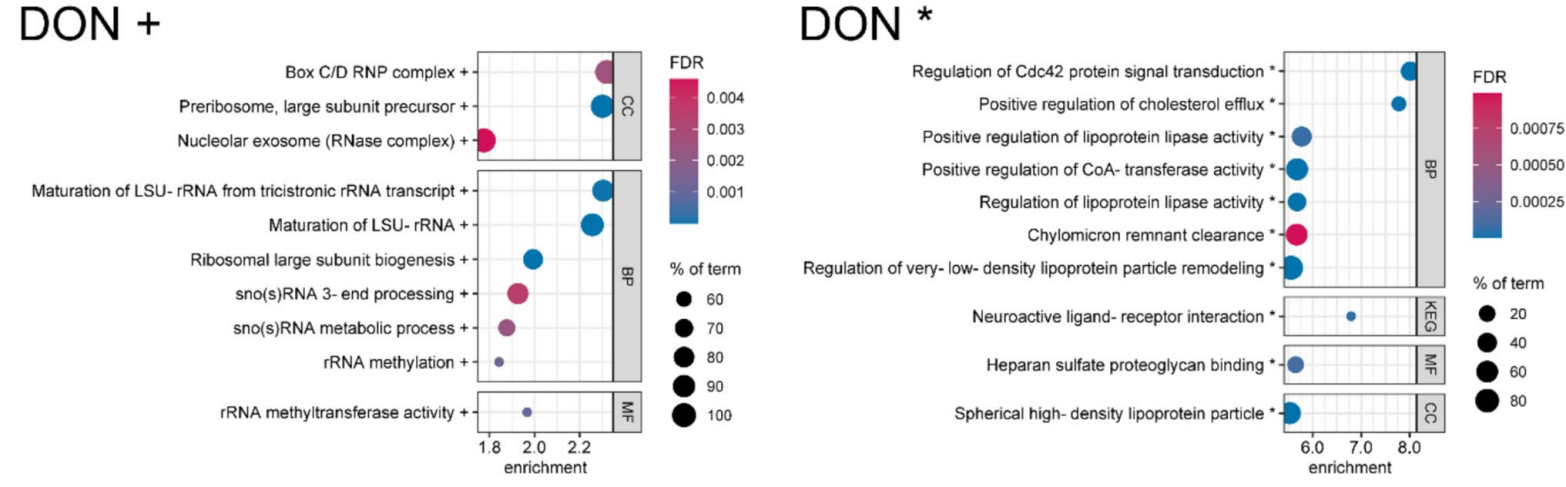
Enrichment bubble plots of the top ten enriched terms for both upregulation (left) and downregulation (right) in the whole proteome of HepG2 cells treated with deoxynivalenol (DON, 1 µM) for 24 h. Effects on cellular components (CC), biological processes (BP), molecular functions (MF, all from Gene Ontology) and terms of the Kyoto Encyclopedia of Genes and Genomes (KEG, in this figure) are shown. The color scale describes the significance as the false discovery rate (FDR) and the bubble size scales with the percentage of identified proteins affiliated to the described term. Terms are annotated by + for upregulation and * for downregulation. Tricistronic rRNA transcript describes the SSU-rRNA, 5.8S rRNA, LSU-rRNA variant. Enrichment analysis was performed by STRING DB

HepG2 cells were treated with 1 µM DON for 24 h. The presented results are divided in enriched terms caused by upregulated (Fig. 4, left) and downregulated proteins (Fig. 4, right). On the upregulated side, the strongest enrichments were observed for the CCs “box C/D RNP^3^ complex” (ES 2.32, FDR 0.0026) and “preribosome, large subunit precursor” (ES 2.30, FDR < 0.0001) and the BPs “maturation of LSU-rRNA^4^” (ES 2.26, FDR < 0.0001), specifically from tricistronic (SSU-rRNA, 5.8S rRNA, LSU-rRNA) rRNA transcript (ES 2.30, FDR < 0.0001). Several further terms described the biogenesis of ribosomes directly or indirectly (MF: “rRNA methyltransferase activity”; BPs: “ribosomal large subunit biogenesis”; “rRNA methylation”). In addition, the CC term “nucleolar exosome (RNAse complex)” and two BP terms on the processing of small nucleolar (sno(s)) RNA were enriched. All these terms were also found enriched in the group of upregulated DAPs (see Online Resource 3) as well as several other terms regarding ribosomes or ribosomal biogenesis.

DON is a well-known ribotoxin and thereby impairs the protein synthesis (McCormick et al. 2011). HepG2 cells seem to counter-regulate the inhibited ribosomal activity by upregulating proteins required to generate new ribosomes. Remarkably, the binding to the LSU becomes apparent in the respective enriched terms, like the “maturation of LSU-rRNA”. Besides the terms directly related to ribosomes, the terms concerning the box C/D RNP complex, the nucleolar exosome and sno(s) RNA are associated with the biogenesis of ribosomes as well, since all these are essential for the maturation of rRNA (Kilchert et al. 2016; Maden and Hughes 1997; Henras et al. 2008).

On the side of downregulated proteins, terms with the strongest enrichments are the BPs “regulation of Cdc42 protein signal transduction” (ES 8.01, FDR < 0.0001) and “positive regulation of cholesterol efflux” (ES 7.78, FDR < 0.0001) as well as the KEGG term “neuroactive ligand-receptor interaction” (ES 6.79, FDR < 0.0001). The latter term included only 1% (3 out of 329) identified proteins of the whole term and was headed by the protein angiotensinogen (AGT, log_2_ FC − 1.91, − log_10_ *p* value 8.28). Remarkably, most terms were influenced by a strong downregulation of different apolipoproteins (Apos, see Online Resource 3). E.g., APOA1 was the strongest downregulated protein of this experiment (log_2_ FC − 2.20, − log_10_ *p* value 3.01). Among some other proteins, the downregulated Apos resulted in an affiliation of most terms to the lipid metabolism. The same was observed for the group of downregulated DAPs, of which mainly Apos led to enriched terms of the lipid metabolism. In addition, some extracellular components and metabolic processes were identified there (see Online Resource 3).

Adverse effects of DON on lipid metabolism were recently described by Jin et al. (2023), who observed disorders in livers of high-fat-diet-induced obesity mice, and by Del Favero et al. (2021), who reported alterations in lipid biosynthesis in human epidermal cells. Previously, weight loss in high-fat-diet-induced mice after DON treatment was also described by Flannery et al. (2013). Our results support these findings, as especially Apos were downregulated. Apos are the protein part of lipoproteins, which represent the transport form of lipids in body fluids. Due to their key role in lipid metabolism, Apo disorders can lead to several illnesses, such as dyslipidemia, obesity or cardiovascular diseases (Albitar et al. 2024). These results led to two hypotheses in order to explain the specific downregulation of Apos. The first one suggests that DON inhibits the cholesterol synthesis comparable to statins, which are drugs used for people with a high risk of cardiovascular diseases (Alenghat and Davis 2019). The decreased cholesterol concentration would finally downregulate the synthesis of Apos. The second hypothesis proposes a link to the ribotoxicity of DON. Potentially, Apos are produced in a very high amount in untreated HepG2 cells and, for that reason, they are the protein class most affected by an inhibited overall protein synthesis in HepG2 cells. Both hypotheses should be investigated in further experiments and could shed light on a second main mechanism of trichothecene toxicity.

### Nivalenol

**Fig. 5:**
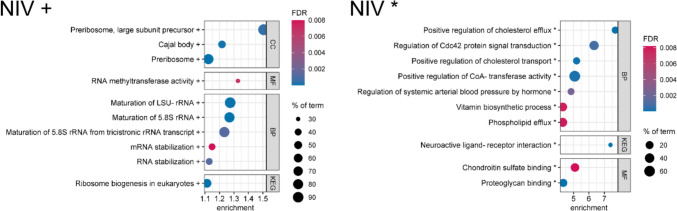
Enrichment bubble plots of the top ten enriched terms for both upregulation (left) and downregulation (right) in the whole proteome of HepG2 cells treated with nivalenol (NIV, 0.5 µM) for 24 h. Effects on cellular components (CC), biological processes (BP), molecular functions (MF, all from Gene Ontology) and terms of the Kyoto Encyclopedia of Genes and Genomes (KEG, in this figure) are shown. The color scale describes the significance as the false discovery rate (FDR) and the bubble size scales with the percentage of identified proteins affiliated to the described term. Terms are annotated by + for upregulation and * for downregulation. Tricistronic rRNA transcript describes the SSU-rRNA, 5.8S rRNA, LSU-rRNA variant. Enrichment analysis was performed by STRING DB

HepG2 cells were treated with 0.5 µM NIV for 24 h. The presented results are divided in enriched terms caused by upregulated (Fig. 5, left) and downregulated proteins (Fig. 5, right). The strongest upregulated terms were observed for the CC “preribosome, large subunit precursor” (ES 1.51, FDR 0.00046) and the MF “RNA methyltransferase activity” (ES 1.33, FDR 0.0071). Comparable to DON, all terms are directly or indirectly associated with the biogenesis of ribosomes, especially of the LSU. The “cajal body” (ES 1.22, FDR < 0.0001) is a ribonucleoprotein particle (RNP) involved in the maturation of spliceosomes and ribosomes (Liang and Li 2011). Again, within the group of upregulated DAPs, mainly ribosome-related terms were found enriched (see Online Resource 3).

The downregulated side is also comparable to DON and dominated by the downregulation of Apos, with APOA1 as the second strongest downregulated protein (log_2_ FC − 1,80, − log_10_ *p* value 1.93). This is supported by the result of enrichment analysis within downregulated DAPs only, which also included extracellular components, the endoplasmic reticulum as well as several metabolism-related terms (see Online Resource 3). However, for NIV, AGT was the strongest downregulated protein (log_2_ FC − 1.89, − log_10_ *p* value 5.16) and was found to mainly affect the KEGG term “neuroactive ligand-receptor interaction” and the BP “regulation of systemic arterial blood pressure by hormone”. Angiotensin, the product of AGT cleavage, is mainly known to regulate blood pressure, but the precursor AGT was also reported to be involved in lipid metabolism, which could explain the co-downregulation with Apos (Kim et al. 2002). The BP “vitamin biosynthetic process” was mainly increased by CYP27A1^5^ and PSAT1^6^ (see Online Resource 3), both of which are involved in vitamin synthesis. As they are not part in the same pathway, this term will not be discussed in detail. Besides the discussed terms, two enriched MFs concerning proteoglycan binding were observed.

The very high overlap between the enriched terms after DON and NIV treatment underlines the similarity in their MoA. This was expected, since both mycotoxins are type B trichothecenes that differ only in the hydroxy group at position 4 for nivalenol yet a hydrogen for deoxynivalenol (Online Resource 1, Figure S1). However, even the small distinction in the chemical structure seems to result in different biological activities, which became apparent in a study on the cytotoxicity in different cell lines, as well: Nagashima (2018) described more than twofold higher concentrations of DON required for 50% inhibition of cell proliferation (IC_50_) compared to NIV.

Previous in vitro bioactivity studies of trichothecenes mainly focused on the inhibition of protein synthesis, apoptosis and inflammation (Rocha et al. 2005). Our bottom-up proteomics approach also characterized the ribotoxicity as a main cellular target and revealed the ability of HepG2 cells to counter-regulate the inhibited protein synthesis in sub-cytotoxic trichothecene concentrations by upregulating the ribosome biogenesis. However, we additionally identified the distinct downregulation of Apos and AGT that could impair the lipid metabolism extensively. Whether the ribotoxicity of DON and NIV is connected to the downregulation of those proteins should be investigated in future studies.

## Conclusion

**Fig. 6:**
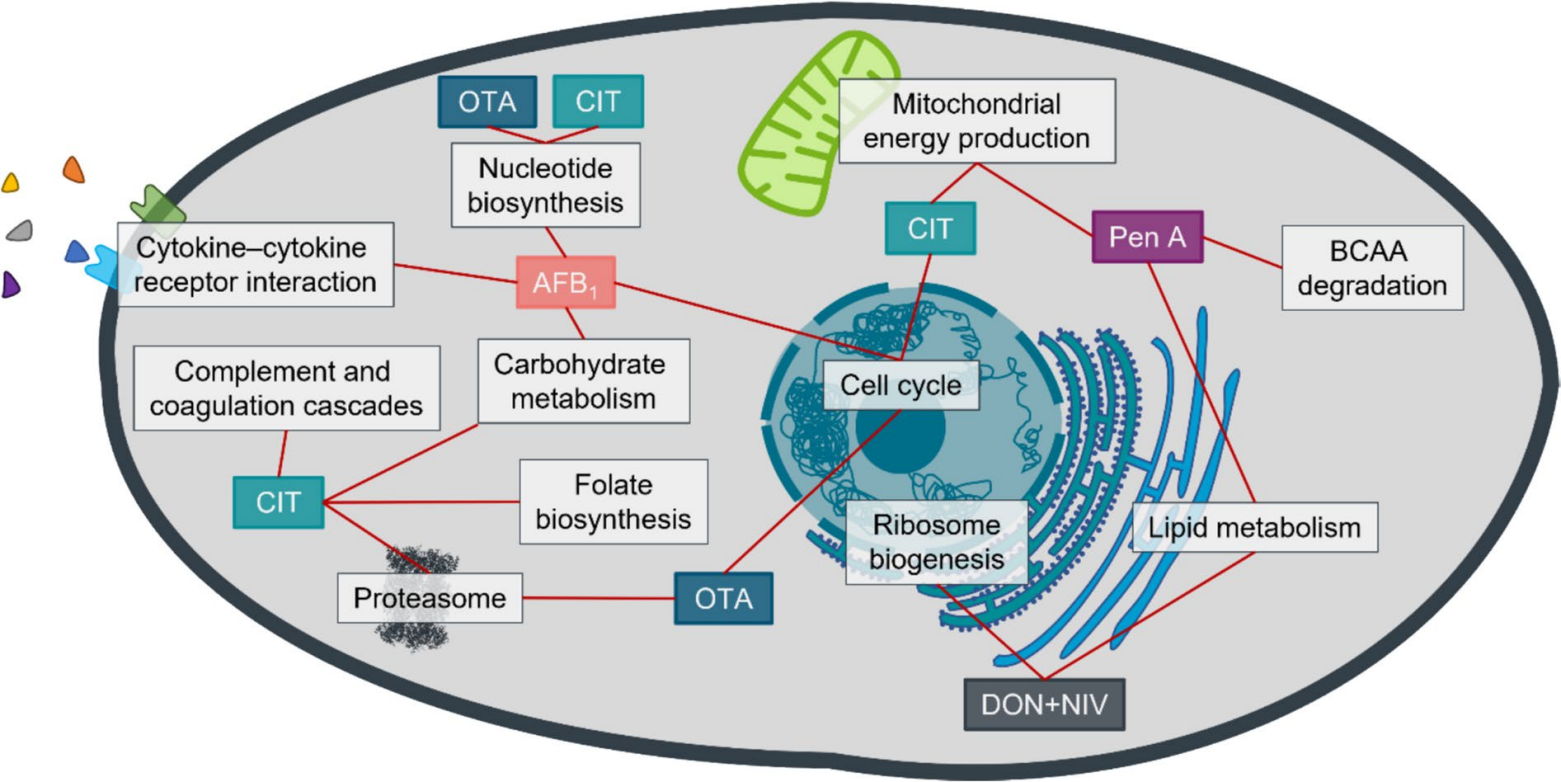
Summarizing overview of the most affected cellular functions of HepG2 and IHKE cells after treatment with selected mycotoxins. AFB_1_: aflatoxin B_1_. CIT: citrinin. DON: deoxynivalenol. NIV: nivalenol. OTA: ochratoxin A. Pen A: penitrem A. BCAA: branched-chain amino acids

The presented work investigated the effects of six selected mycotoxins on the proteomes of human hepatoblastoma cells (HepG2) and human epithelial kidney cells (IHKE). The aim was the identification of main cellular targets and the underlying MoA. The cells were treated with sub-cytotoxic concentrations of the mycotoxins to induce proteomic alterations without activating acute cell death mechanisms directly. An overview of the effects on the cellular proteomes is depicted in Fig. 6. For instance, the trichothecenes DON and NIV induced a specific upregulation of proteins that are involved in the biogenesis of ribosomes. In the strongest enriched terms, the binding of these mycotoxins to the LSU became apparent. On the downregulated side, certain terms regarding the lipid metabolism were enriched, mainly driven by decreased Apos. Moreover, OTA and CIT revealed some commonalities as well, inducing the upregulation of the MCM complex and nucleotide biosynthesis, presumably indicating replication stress. The shared proteasome upregulation by OTA and CIT could be a more unspecific response towards oxidative stress or indicate a direct interaction of these mycotoxins with proteins. The effects on DNA replication, proteasome and nucleotide synthesis suggest a similarity between the MoA of OTA and CIT that could be caused by their coumarin-derived backbone. However, CIT seems to affect also primary metabolic pathways such as fructose, mannose and folate metabolism. AFB_1_ induced the upregulation of GDF15 and some cytokine receptors, pointing towards an inflammatory response. Within the upregulated DAPs, a specific effect on mitosis and cytokinesis was identified. Furthermore, after pretreatment of HepG2 cells with β-NF to induce metabolic activity and thereby generate AFB_1_ phase I metabolites in vitro, several proteins involved in ribosome biogenesis were specifically downregulated, which was reflected in respective terms. Pen A affected mainly the sterol metabolism, but showed further effects on the mitochondrial energy production and branched-chain amino acid degradation. The effects of CIT and OTA on IHKE proteomes were different to those in HepG2, but still supported their proposed MoA in terms of replication stress.

In conclusion, the investigated mycotoxins caused diverse responses on the cellular proteomes. On the one hand, some well-known toxicity pathways were observed as strongly affected biological functions, e.g., in the form of ribosome biogenesis upregulated by trichothecenes or inflammatory response after AFB_1_ treatment. On the other hand, novel potential targets were identified, like the cholesterol metabolism affected by Pen A, ribosomal proteins and cell cycle affected by AFB_1_, Apos modulated by trichothecenes and alterations in DNA replication proteins induced by OTA and CIT.

Certainly, the presented study exhibits some limitations. Approximately, 3000 proteins were quantified in the datasets that evidently only represent a part of the proteome. The described effects were proposedly observed in abundant proteins, which limits the overall completeness of proteomic analyses in general. Any alterations within the lowest abundant proteins are not captured by such methods. For this purpose, more powerful instruments or deep proteome approaches are required. In addition, results from in vitro experiments only allow a limited prediction of the in vivo situation, as several toxicokinetic and -dynamic factors are not taken into account.

However, the presented results still enable the identification of cellular effects of mycotoxins, caused by well described as well as by potentially new MoA. Therefore, the study evidently encompasses novel aspects in terms of mycotoxins’ cellular targets that should improve the elucidation of their toxicodynamic properties. Thus, this work represents a significant progress for mycotoxin research within the AOP framework. Furthermore, it underlines the high potency of omics techniques to characterize biological activities of compounds of interest in an unprecedented way.

Future studies should focus on a more detailed elucidation of the proposed effects. This could be accomplished for example by investigating concentration and time dependence. As the method confirmed previously described MoA, it can be applied to investigate the effects of less well-characterized mycotoxins and also be transferred to other types of cells or even ex vivo samples. Furthermore, single mechanisms could be analyzed in highly specific assays. This would signify a huge step towards a deeper elucidation of the bioactivity of mycotoxins.

## Supplementary Information

Below is the link to the electronic supplementary material.

Supplementary file1 (DOCX 152 KB)

Supplementary file2 (XLSX 147 KB)

Supplementary file3 (XLSX 15405 KB)

Supplementary file4 (XLSX 6611 KB)

## Data Availability

All relevant data can be found in the Electronic Supplementary Material (ESM). Online Resource 1 contains detailed information on the HPLC–MS method, the chemical structures of the investigated mycotoxins and one figure about the percentage of differentially abundant proteins (DAPs) of each experiment. Online Resource 2 contains statistical background proteomes of the two cell lines, used for interaction analysis of DAPs in STRING DB. Online Resources 3 and 4 contain abundance and significance data of single proteins and enriched biological functions within groups of deregulated proteins (interaction and enrichment analysis) of each HepG2 and IHKE experiment, respectively.

## Footnotes

1 Minichromosome maintenance.

2 Cell division cycle 45–minichromosome maintenance–Go-Ichi-Ni-San.

3 Ribonucleoprotein particles.

4 Ribosomal ribonucleic acid.

5 Cytochrome P450 monooxygenase 27A1.

6 Phosphoserine aminotransferase 1.
