# Supplementary material for: Alterations in the proteomes of HepG2 and IHKE cells inflicted by six selected mycotoxins
Source: Arch Toxicol. 2024 Dec 6;99(2):701–15. doi: 10.1007/s00204-024-03905-0 (PMC11775057; doi:10.1007/s00204-024-03905-0)
Supplement: Supplementary file 1 — Supplementary file1 (DOCX 152 KB) [file 204_2024_3905_MOESM1_ESM.docx]

Supplementary material

for

Six selected mycotoxins inflict adverse alterations on the proteome of HepG2 and IHKE cells

Lucas Keuter, Marco Fortmann, Matthias Behrens, Hans-Ulrich Humpf * (humpf@uni-muenster.de)

Institute of Food Chemistry, University of Münster, Corrensstraße 45, 48149 Münster, Germany

Journal: Archives of Toxicology

# Materials and methods

**Chemicals and reagents**

The solvents were purchased in HPLC-MS grade from Carl Roth (Karlsruhe, Germany), Fisher Scientific (Schwerte, Germany) or Sigma-Aldrich (Steinheim, Germany). Purified water was generated using a PureLab Flex2 system (Veolia Water Technologies, Celle, Germany). Sequencing grade trypsin was purchased from Serva (Heidelberg, Germany). Mycotoxins were obtained from the following sources: Pen A, ≥95% and CIT, ≥98% from Enzo Life Sciences (Farmingdale, NY, USA); DON, ≥95% provided from Bretz et al. 2006; NIV, ≥98% from Sigma-Aldrich; AFB_1_, ≥98% from Acros Organics (Geel, Belgium); OTA, ≥99% provided from Sueck et al. 2019. If not specified otherwise, all other chemicals were purchased from Sigma-Aldrich or Carl Roth.

**Cell culture**

The human hepatoblastoma (López-Terrada et al. 2009) cell line HepG2 (RRID: CVCL_0027, ATCC, Manassas, VA, USA) was cultured in Dulbecco’s Modified Eagle’s Medium (Gibco™ DMEM, high glucose, with glutamine, Thermo Fisher Scientific, Darmstadt, Germany) supplemented with 10 mM *N*‑2‑hydroxyethylpiperazine‑*N*’‑2-ethane-sulfonic acid (HEPES buffer), 10% fetal calf serum (FCS, Gibco™, Thermo Fisher Scientific, Darmstadt, Germany) and 100 U/mL penicillin and 100 µg/mL streptomycin (PAN Biotech, Aidenbach, Germany) as antibiotics. The cells were subcultured after trypsinization once a week and the cell culture medium was replaced twice a week. The immortalized human kidney epithelial cell line IHKE (Tveito et al. 1989; Rottkord et al. 2017; RRID: CVCL_0344, kindly provided by S. Mollerup, National Institute of Occupational Health, Norway) was cultured in Gibco™ DMEM (high glucose, with glutamine) supplemented with 15 mM HEPES, 1% FCS, 5 mg/L of each insulin and transferrin, 5 µg/L selenite (as sodium selenite), 10 µg/L epidermial growth factor and 500 µg/L hydrocortisone. The cells were subcultured after trypsinization twice a week and the culture medium was exchanged one day after every subculturing. Both cell lines were cultured at 37 °C in humidified atmosphere with 5% CO_2_.

**Sample preparation**

Proteomic sample preparation was performed according to the filter-aided sample preparation (FASP) protocol of Wiśniewski et al. and as described previously (Wiśniewski et al. 2009; Müller et al. 2022). After incubation, medium was removed and cells were rinsed twice with cold phosphate-buffered saline (PBS). After scraping, cells were pelleted and lysed by ultrasonic treatment in sodium dodecylsulfate-based lysis buffer with dithiothreitol. The samples were heated to 95 °C, before cell debris was removed by centrifugation. Protein amount of HepG2 lysates was determined by tryptophane fluorescence (Wiśniewski and Gaugaz 2015). Therefore, excitation at 295 nm and emission at 350 nm were set in a TECAN infinite M200Pro microplate reader (TECAN, Crailsdorf, Germany). Protein amount of IHKE lysates was determined by bicinchonic acid (BCA) assay using Pierce™ BCA reagent according to the manufacturer’s protocol (Smith et al. 1985). Bovine serum albumin was used as reference for determination of protein amounts. Cell lysate supernatant containing 300 µg of protein was diluted in urea buffer in a Microcon^®^ filter unit (Microcon-30 kDa, Merck Millipore, Billerica, MA, USA), followed by centrifugation. Alkylation was performed by using iodoacetamide. After three washing steps with urea buffer and ammonium bicarbonate buffer each, proteins were digested with trypsin (trypsin:protein ratio 1:100) in a wet chamber under shaking at 37 °C overnight. Peptides were eluted, acidified, desalted by solid-phase extraction, evaporated and reconstituted in 100 µL ACN/water (1+19, *V*/*V*).

Table S1. HPLC-MS parameter for proteomics of cell digests. The HPLC-MS method was modified based on the Bruker default application method for shotgun proteomics on an Impact™ II instrument. Selected parameters are depicted here.

| **Software** | | | | Compass® HyStar v 5.1, otofControl v 6.3, DataAnalysis v 5.1 | | | | | | | | | |
| --- | --- | --- | --- | --- | --- | --- | --- | --- | --- | --- | --- | --- | --- |
| **Instrument** | | | | Bruker Elute HT Pump HPG 1300, CTC Analytics PAL HTC-xt,  Bruker Elute column oven, Bruker Impact™ II with an Apollo™ II source | | | | | | | | | |
| **Column** | | | | Agilent AdvanceBio Peptide Mapping 120 Å, 2.1 x 150 mm, 2.7 µm equipped with a Phenomenex KrudKatcher Classic 0.5 µm inline filter | | | | | | | | | |
| **Column oven** | | | | 40 °C | | | | | | | | | |
| **Gradient** flow rate: 200 µL/min | | | | min | | | | % ACN + 0.1% FA | | | | % H_2_O + 0.1% FA | |
|  |  |  |  | 0 | | | | 8 | | | | 92 | |
|  |  |  |  | 3 | | | | 8 | | | | 92 | |
|  |  |  |  | 90 | | | | 29 | | | | 71 | |
|  |  |  |  | 95 | | | | 60 | | | | 40 | |
|  |  |  |  | 95.1 | | | | 100 | | | | 0 | |
|  |  |  |  | 100 | | | | 100 | | | | 0 | |
|  |  |  |  | 100.1 | | | | 8 | | | | 92 | |
|  |  |  |  | 110 | | | | 8 | | | | 92 | |
| **Calibrant** | | | | Agilent Tuning Mix for ESI-TOF (part nr G1969-85000) | | | | | | | | | |
|  | | **Scan range** | | 150–2200 *m*/*z* | | | **Spectra rate MS^1^** | | | | 2 Hz | | |
| **Source** | | **Polarity** | | positive | | | **Nebulizer gas** | | | | 4 bar | | |
|  |  | **End plate offset** | | 500 V | | | **Dry gas** | | | | 12 L/min | | |
|  |  | **Capillary voltage** | | 4500 V | | | **Dry temperature** | | | | 250 °C | | |
| **Tune** | | **Funnel 1 RF** | | 400 Vpp | | | **Collision energy** | | | | 7 eV | | |
|  |  | **Funnel 2 RF** | | 600 Vpp | | | **Transfer time** | | | | 90 µs | | |
|  |  | **Hexapole** | | 350 Vpp | | | **Collision RF** | | | | 2000 Vpp | | |
|  |  | **Quadrupole** | | 5 eV | | | **Pre pulse storage** | | | | 10 µs | | |
|  |  | **Low mass** | | 300 *m*/*z* | | |  | | | |  | | |
| **MS/MS** | | **Scan mode** | | Auto MS/MS | | | **Active exclusion** | | | | On | | |
|  |  | **Cycle time** | | 3 s | | | **Exclude after** | | | | 1 spectrum | | |
|  |  | **Threshold** | | 2500 | | | **Release after** | | | | 0.5 min | | |
|  |  | **Smart exclusion** | | 9 x | | | **Reconsider precursor, if** | | | | Current/ previous Intensity = 3 | | |
| **Preference** | | **Charge state** | | 2–5 | | | **Group length** | | | | 5 | | |
|  |  | **Exclude singly** | | On | | | **Strict active exclusion** | | | | Off | | |
|  |  | **Exclude unknown** | | On | | |  | | | |  | | |
| **AcqCtrl** | | **Acquisition control** | | Dynamic | | | **Max. MS/MS acq.** | | | | 32 Hz | | |
|  |  | **Target intensity** | | 7000 | | | **Min. MS/MS acq.** | | | | 2 Hz | | |
| Collision induced dissociation | Mass | | Width | Collision | Charge state | Mass | | | Width | Collision | | | Charge state |
|  | 400 | | 2 | 23 | 1 | 800 | | | 3 | 40 | | | 3 |
|  | 400 | | 2 | 23 | 2 | 900 | | | 5 | 50 | | | 1 |
|  | 400 | | 2 | 23 | 3 | 900 | | | 5 | 50 | | | 2 |
|  | 500 | | 3 | 27 | 1 | 900 | | | 5 | 45 | | | 3 |
|  | 500 | | 3 | 27 | 2 | 1000 | | | 5 | 55 | | | 1 |
|  | 500 | | 3 | 25 | 3 | 1000 | | | 5 | 55 | | | 2 |
|  | 600 | | 3 | 33 | 1 | 1000 | | | 5 | 50 | | | 3 |
|  | 600 | | 3 | 33 | 2 | 1100 | | | 5 | 65 | | | 1 |
|  | 600 | | 3 | 27 | 3 | 1100 | | | 5 | 65 | | | 2 |
|  | 700 | | 3 | 33 | 1 | 1100 | | | 5 | 55 | | | 3 |
|  | 700 | | 3 | 33 | 2 | 1300 | | | 5 | 65 | | | 1 |
|  | 700 | | 3 | 27 | 3 | 1300 | | | 5 | 65 | | | 2 |
|  | 800 | | 3 | 45 | 1 | 1300 | | | 5 | 55 | | | 3 |
|  | 800 | | 3 | 45 | 2 | Fallback charge state | | | | | | | 3 |

Figure S 2. Percentage of differentially abundant proteins (DAPs) per experiment. Pen A: penitrem A, 10 µM. AFB1: aflatoxin B_1_, 10 µM. b-NF: β-naphtoflavone. CIT: citrinin, 20 µM in HepG2, 15 µM in IHKE. OTA: ochratoxin A, 200 nM in HepG2, 20 nM in IHKE. DON: deoxynivalenol, 1 µM. NIV: nivalenol, 0.5 µM. Each treatment was carried out for 24 h. Count of DAPs was determined by Perseus software.

Figure S 1. Chemical structures of selected mycotoxins.

References

Bretz M, Beyer M, Cramer B, Knecht A, Humpf H-U (2006) Thermal degradation of the Fusarium mycotoxin deoxynivalenol. J Agric Food Chem 54(17):6445–6451. https://doi.org/10.1021/jf061008g

López-Terrada D, Cheung SW, Finegold MJ, Knowles BB (2009) Hep G2 is a hepatoblastoma-derived cell line. Hum Pathol 40(10):1512–1515. https://doi.org/10.1016/j.humpath.2009.07.003

Müller L, Keuter L, Bücksteeg D, et al. (2022) Metabolic conjugation reduces in vitro toxicity of the flavonoid nevadensin. Food Chem Toxicol 164:113006. https://doi.org/10.1016/j.fct.2022.113006

Rottkord U, Röhl C, Ferse I, et al. (2017) Structure-activity relationship of ochratoxin A and synthesized derivatives: importance of amino acid and halogen moiety for cytotoxicity. Arch Toxicol 91(3):1461–1471. https://doi.org/10.1007/s00204-016-1799-3

Smith PK, Krohn RI, Hermanson GT, et al. (1985) Measurement of protein using bicinchoninic acid. Anal Biochem 150(1):76–85. https://doi.org/10.1016/0003-2697(85)90442-7

Sueck F, Hemp V, Specht J, Torres O, Cramer B, Humpf H-U (2019) Occurrence of the Ochratoxin A Degradation Product 2'R-Ochratoxin A in Coffee and Other Food: An Update. Toxins (Basel) 11(6). https://doi.org/10.3390/toxins11060329

Tveito G, Hansteen I-L, Dalen H, Haugen A (1989) Immortalization of Normal Human Kidney Epithelial Cells by Nickel(II)1. Cancer Res 49(7):1829–1835

Wiśniewski JR, Gaugaz FZ (2015) Fast and sensitive total protein and Peptide assays for proteomic analysis. Anal Chem 87(8):4110–4116. https://doi.org/10.1021/ac504689z

Wiśniewski JR, Zougman A, Nagaraj N, Mann M (2009) Universal sample preparation method for proteome analysis. Nat Methods 6(5):359–362. https://doi.org/10.1038/nmeth.1322
